# How do plants maintain pH and ion homeostasis under saline-alkali stress?

**DOI:** 10.3389/fpls.2023.1217193

**Published:** 2023-10-17

**Authors:** Jing Li, Yongqing Yang

**Affiliations:** ^1^ Key Laboratory for Northern Urban Agriculture of Ministry of Agriculture and Rural Affairs, College of Bioscience and Resources Environment, Beijing University of Agriculture, Beijing, China; ^2^ College of Biological Sciences, China Agricultural University, Beijing, China

**Keywords:** saline-alkali stress, ion homeostasis, pH homeostasis, plant cell, abiotic stress

## Abstract

Salt and alkaline stresses often occur together, severely threatening plant growth and crop yields. Salt stress induces osmotic stress, ionic stress, and secondary stresses, such as oxidative stress. Plants under saline-alkali stress must develop suitable mechanisms for adapting to the combined stress. Sustained plant growth requires maintenance of ion and pH homeostasis. In this review, we focus on the mechanisms of ion and pH homeostasis in plant cells under saline-alkali stress, including regulation of ion sensing, ion uptake, ion exclusion, ion sequestration, and ion redistribution among organs by long-distance transport. We also discuss outstanding questions in this field.

## 1 Introduction

Plants face many biotic and abiotic stresses from their environment. Soil salinization and alkalization cause widespread abiotic stress in plants, adversely affecting global crop productivity. Saline-alkali stress includes the combined effects of salt and alkali stresses.

Salt stress is a major threat limiting plant growth and crop yield (Yang and Guo, 2018a; Kotula et al., 2020). More than 800 million hectares of the world’s land are affected by salinity (Munns and Tester, 2008). One-fifth of irrigated lands are already affected (Morton et al., 2019), and this area is growing larger as a result of overirrigation and climate changes. Soils are classified as saline when the electrical conductivity of a saturated soil extract is above 4 dS·m^–1^. Halophytic plants tolerate high salt concentrations, while glycophytes are sensitive to saline soils (Munns and Tester, 2008; Flowers et al., 2015). Most crop species are glycophytes, and their growth and reproduction are severely affected by salt stress, which is typically caused by sodium chloride, sodium sulfate, and other neutral salts in soil. Sodium ions (Na^+^) are generally considered to be the major toxic ions at high concentrations. Three stress signaling pathways are induced under salt stress: osmotic stress, ionic stress, and secondary stress (Sewelam et al., 2014; Yang and Guo, 2018a). High salt concentrations increase the osmotic pressure and lower the water potential of soil solutions, leading to water deficiency in plants (Hasegawa et al., 2000). Na^+^ in plant cells affect the absorption of other ions necessary for normal physiological processes (Yang and Guo, 2018b). Osmotic stress and ionic stress cause long-term secondary stresses, including oxidative stress. Salt-induced oxidative stress leads to accumulation of reactive oxygen species (ROS) in plant cells, including singlet oxygen (^1^O_2_), superoxide anion (O_2_^–^), hydrogen peroxide (H_2_O_2_), and hydroxyl radicals (HO·). Excess ROS cause oxidative damage to DNA, proteins, and lipids, affecting the integrity of plant cell organelles.

Alkali stress is often concomitant with salt stress owing to the abundance of sodium carbonate and sodium bicarbonate in soil. High pH in soil causes deposition of metal ions, such as iron (Fe^2+^), magnesium (Mg^2+^), and calcium (Ca^2+^). The uptake of mineral elements is blocked in plants, leading to nutrient deficiency. Meanwhile, many plant metabolic activities are severally interrupted, such as root vitality, cell pH stability, cell membrane integrity, and photosynthesis.

The combination of saline and alkali stresses affects plant growth and development more severely than salt stress or alkali stress alone. Most studies have focused mainly on either salt stress or alkali stress, with only a few paying attention to mixed saline-alkali stress. With an increasing world population, a 50% increase in food production by 2050 will be required (FAO, 2017). Understanding the mechanisms of plant responses to saline-alkali stress will provide information for genetically engineering saline-alkali-tolerant crops to meet this demand. Here, we discuss recent studies on the mechanisms underlying plant responses to saline-alkali stress.

## 2 Sensing saline-alkali stress

High concentrations of salt and high soil pH induce multiple stresses in plant cells. Plants must sense saline-alkali stress rapidly and trigger precise responses. Sensing Na^+^, osmotic stress, and high pH is the first step in initiating such responses. Salt-induced Na^+^ accumulation occurs within 2 minutes after exposure to salt stress, while Na^+^ begins to efflux from roots within 10 minutes (Essah et al., 2003; Bose et al., 2015). This indicates that high Na^+^ concentrations are rapidly perceived, triggering specific downstream responses.

The mechanism underlying salt stress sensing has been an open question for a long time. High salt induces both ionic and osmotic stresses in plants. It was thought that plants perceive osmotic stress rather than Na^+^ (Munns and Tester, 2008). However, rapid sodium-specific calcium waves have since been identified (Dodd et al., 2010; Hashimoto and Kudla, 2011; Martí et al., 2013). Salt (NaCl) induces an increase in the cytosolic free calcium concentration ([Ca^2+^]_cyt_) in cortical and endodermal cells of roots, while osmotic stress (mannitol) induces an increase in [Ca^2+^]_cyt_ in epidermal cells (Kiegle et al., 2000; Choi et al., 2014). Increased [Ca^2+^]_cyt_ activates Ca^2+^-binding proteins and upregulates the Na^+^/H^+^ antiporter to efflux Na^+^. Accordingly, plants may perceive salt stress through Na^+^ sensors and osmotic sensors in different cell types.

### 2.1 Osmotic sensor

Osmotic sensor contributes to the perception of salt stress. The Arabidopsis (*Arabidopsis thaliana*) *reduced hyperosmolality-induced [Ca^2+^] increase 1 (osca1)* mutant was isolated through Ca^2+^-imaging-based forward genetic screening (Yuan et al., 2014). Under osmotic stress, OSCA1 induces a rapid increase in [Ca^2+^]_cyt_. OSCA1 is a Ca^2+^-permeable channel on the plasma membrane identified as a putative sensor of hyperosmotic stress and required for osmotic-stress-induced Ca^2+^ signaling (Yuan et al., 2014). The *osca1* mutant shows impaired Ca^2+^ signaling in response to osmotic stress but not salt stress (Yuan et al., 2014). Recent mathematical fitting/modeling analysis in wild-type Arabidopsis and the *osca1* mutant revealed that OSCA1 is responsible for the increase of [Ca^2+^]_cyt_ induced by osmotic stress rather than ionic stress (Pei et al., 2022).

### 2.2 Ionic sensor

The plant cell wall is involved in perceiving and responding to salt stress (Endler et al., 2015; Van der Does et al., 2017; Parrotta et al., 2015). It is important for cells to sense salt-induced changes in the cell wall and maintain cell wall integrity. Plasma membrane-located receptor-like kinases (RLKs), MALE DISCOVERER 1-INTERACTING RECEPTOR LIKE KINASE 2 (MIK2), FEI1 and FEI2 (named after the Chinese word for fat), and FERONIA (FER) are involved in cell wall sensing and the response to salt stress (Xu et al., 2008; Van der Does et al., 2017; Feng et al., 2018). Salt-specific ionic stress causes cell wall softening, which is sensed by FER (Feng et al., 2018). FER interacts with pectin in the cell wall and elicits salt-induced Ca^2+^ transients to maintain cell wall integrity under high salinity. Thus, FER senses cell wall integrity under salt stress. Salt stress also induces the secretion of mature RAPID ALKALINIZATION FACTORs (RALFs), which interact with FER to trigger a series of physiological process such as deactivation of plasma membrane H^+^-ATPases (PM H^+^-ATPases) and apoplastic alkalinization (Haruta et al., 2014).

The *Arabidopsis monocation-induced Ca^2+^ increases 1* (*moca1*) mutant was identified through a Ca^2+^-imaging-based forward genetic screen for mutants with disturbed [Ca^2+^]_cyt_ induced by salt. Increases in [Ca^2+^]_cyt_ in response to Na^+^, potassium ions (K^+^), or lithium ions (Li^+^) are reduced in the *moca1* mutant than in wild-type plants (Jiang et al., 2019). Phenotype analysis revealed that the *moca1* mutant is hypersensitive to salt stress. MOCA1 is a glucuronosyltransferase for glycosylinositol phosphorylceramide (GIPC) sphingolipids in the plasma membrane. Na^+^ binds to GIPC to potentially gate Ca^2+^ channels mediating Ca^2+^ influx and induce downstream responses. Therefore, MOCA1-dependent GIPC sphingolipids are likely involved in sensing salt stress through unidentified Ca^2+^ transporters in the plasma membrane (Jiang et al., 2019). Arabidopsis ANNEXIN1 (ANN1) and AtANN4 are Ca^2+^-permeable transporters (Laohavisit et al., 2013; Ma et al., 2019) required for salt-induced increases in [Ca^2+^]_cyt_. AtANN4 interacts with SALT OVERLY SENSITIVE 2 (SOS2) and SOS3-LIKE CALCIUM-BINDING PROTEIN 8 (SCaBP8) to generate Ca^2+^ signals that activate the salt overly sensitive (SOS) pathway under salt stress. Meanwhile, AtANN4 is repressed by the SOS pathway, forming a negative feedback loop to fine-tune Ca^2+^ signals for optimizing the plant salt response (Ma et al., 2019). It remains to be confirmed whether the MOCA1-dependent GIPC sphingolipids gate operates through AtANN1 and AtANN4 to trigger downstream responses to salt -specific ionic stress.

Different from animals, plants might sense salt through a multicomponent ion channel complex. In addition, this ion channel complex might not be controlled directly by salt (Jiang et al., 2019; van Zelm et al., 2020; Pei et al., 2022). Identifying the salt sensor remains a problem that scientists are eager to solve.

### 2.3 pH sensor

Alkali stress is often associated with salt stress. However, high pH may be sensed through a different mechanism in plants. The cell-surface peptide-receptor complexes RGF1-RGFR (involving ROOT MERISTEM GROWTH FACTOR 1 [RGF1] and its receptors [RGFRs]) and Pep1-PEPR (involving plant elicitor peptides [Peps] and their receptors [PEPRs]) function as pH sensors to regulate extracellular pH-mediated growth and immunity in root apical meristem regions (Liu et al., 2022). Extracellular alkalization inhibits the interaction between RGF1 and RGFRs through sulfotyrosine, thereby inhibiting root meristem growth. Extracellular alkalization also facilitates alkaline-dependent binding of Peps to PEPRs through the pH sensor Glu/Asp, thereby promoting immunity (Liu et al., 2022). Further investigation is needed to reveal whether high pH during alkali stress is also sensed by plant membrane RLKs.

### 2.4 Bicarbonate sensor

In addition to high pH, excess bicarbonate or carbonate caused by saline-alkali stress may injury plants in unique way. *SLOWLY ACTIVATING ANION CONDUCTANCE HOMOLOGUE 3* in *Glycine soja* (*GsSLAH3*) was found to be induced be NaHCO_3_ treatment. Overexpression of *GsSLAH3* in Arabidopsis improves alkaline tolerance under treatments of NaHCO_3_ or KHCO_3_, rather than high pH treatment, which indicates that *GsSLAH3* is a positive alkali responsive gene that increases bicarbonate resistance specifically (Duan et al., 2018). Similarly, GsERF6 and GsBOR2 also exhibit specific response to bicarbonate stress (Yu et al., 2016; Duan et al., 2018). These results suggest that plants response to bicarbonate stress in a unique way. It is still indistinct whether plants also possess unique sensors to bicarbonate stress.

## 3 Importing ions into plant cells

It is important to maintain ion homeostasis in plant cells under saline-alkali stress. Under normal conditions, plants maintain relatively high K^+^ concentrations and low Na^+^ concentrations in cells (Binzel et al., 1988). A high ratio of cytosolic K^+^ to Na^+^ is necessary for plant growth and development (Hussain et al., 2021). However, under saline-alkali stress, excess Na^+^ is transported into the cytoplasm, which disturbs ion homeostasis.

Plants maintain Na^+^ homeostasis under saline-alkali stress by restricting Na^+^ import, compartmentalizing Na^+^, and increasing Na^+^ efflux (Yamaguchi et al., 2013; Yang and Guo, 2018a). The specific import system for Na^+^ has not been identified, but Na^+^ is known to be transported into cells through K^+^ carriers. Electrophysiological evidence shows that Na^+^ is transported across the plasma membrane through nonselective cation channels (NSCCs), high-affinity K^+^ transporters (HKTs), and low-affinity K^+^ transporters (such as ARABIDOPSIS K^+^ TRANSPORTER 1 [AKT1]) (Blumwald et al., 2000; Tuteja, 2007).

HKTs are key determinants of plant responses to salt stress (Horie et al., 2006; Platten et al., 2006; Horie et al., 2007). HKT1 from wheat (*Triticum aestivum*) was first identified as an H^+^/K^+^ symporter and then confirmed as a Na^+^/K^+^ symporter (Schachtman and Schroeder, 1994; Rubio et al., 1995). High-affinity K^+^ uptake by HKT1 is activated by micromolar concentrations of Na^+^ and inhibited by high concentrations of Na^+^, which initiate low-affinity Na^+^ uptake (Rubio et al., 1995). AtHKT1 from Arabidopsis and OsHKT1 from rice (*Oryza sativa*) display selective Na^+^ transport activity rather than K^+^ transport activity in Xenopus (*Xenopus laevis*) oocytes (Uozumi et al., 2000; Horie et al., 2001; Garciadeblás et al., 2003). OsHKT1 plays an exclusive role in Na^+^ uptake by rice roots under K^+^ starvation. However, under salt stress, OsHKT1 activity is rapidly downregulated to restrict excess Na^+^ influx (Horie et al., 2007). Arabidopsis *hkt1* mutants accumulate more Na^+^ than wild-type plants and are sensitive to salt stress (Mäser et al., 2002; Rus et al., 2004; Davenport et al., 2007). Excess Na^+^ influx into the cytoplasm can decrease membrane potential. Therefore, K^+^ outflow channels (NSCC, STELAR K^+^ OUTWARD RECTIFIER [SKOR], and GATED OUTWARDLY-RECTIFYING K^+^ CHANNEL [GORK]) are activated and the ratio of K^+^ to Na^+^ decreased, weakening plant adaption to salt stress (Falhof et al., 2016). Recently, AKT1 is found to mediates K^+^ uptake under salt stress (Li et al., 2023). SCaBP8 binds to AKT1 to reduce its activity (Ren et al., 2013); under salt stress, phosphorylation of SCaBP8 by SOS2, promoted by phosphatidic acid (PA), releases the inhibition of AKT1, enhancing K^+^ influx (Li et al., 2023). Meanwhile, PA also promotes Na^+^ efflux through the SOS2–SOS1 module to maintain Na^+^/K^+^ ratios under salt stress (Li et al., 2023).

In addition to salt-induced Na^+^ influx and changes in the Na^+^/K^+^ ratio under saline-alkali stress, high pH also causes nutrient ions, such as Ca^2+^, Mg^2+^, Fe^2+^, zinc (Zn^2+^), and copper (Cu^2+^), to precipitate in soil. Under saline-alkali stress, nutrient recycling and availability for root cells are decreased, further leading to nutrient deficiency in plants (Jia et al., 2020). Saline-alkali stress induces plants to generate and secrete organic acids such as oxalate, acetate, and malate, which function as buffering substances to maintain intracellular pH homeostasis and ion balance (Fang et al., 2021). The key enzyme in this biochemical process is phosphoenolpyruvate carboxylase, which is regulated by phosphoenolpyruvate carboxylase kinases. These kinases are dramatically upregulated by NaHCO_3_ stress (Xiang et al., 2019).

## 4 Na^+^ exclusion

Excess Na^+^ accumulates in plant cytoplasm under saline-alkali stress. Plants transport Na^+^ from the cytoplasm to the apoplast or vacuoles mainly through Na^+^/H^+^ antiporters, which transport Na^+^ in exchange for H^+^ (Mansour and Hassan, 2022).

The signaling pathways for Na^+^ efflux have been well described, with the most important being the SOS regulatory pathway (Yang and Guo, 2018a; Yang and Guo, 2018b; Ismail et al., 2020; van Zelm et al., 2020). The SOS pathway has three major components: SOS1, SOS2, and SOS3 or SCaBP8. SOS1 is a Na^+^/H^+^ antiporter belonging to the Na^+^/H^+^ exchangers (NHX) family. SOS2 is a serine/threonine-protein kinase. SOS3 and SCaBP8 are EF-hand calcium-binding proteins. SOS3 mainly functions in roots while SCaBP8 mainly functions in shoots (Quan et al., 2007; Monihan et al., 2016; Yang and Guo, 2018a). *SCaBP8* overexpression in *sos3* mutants partially rescues the *sos3* sensitive phenotype under salt stress. However, *SOS3* overexpression fails to complement the *scabp8* mutant. These results indicate that SCaBP8 and SOS3 are only partially redundant in their function, with each playing additional and unique roles in the plant response to salt stress (Quan et al., 2007). Under salt stress, SOS3 and SCaBP8 perceive salt-induced [Ca^2+^]_cyt_ to release self-inhibition of SOS2, recruiting SOS2 to the plasma membrane and activating SOS2 (Halfter et al., 2000; Kim et al., 2007; Lin et al., 2009). SOS2 then phosphorylates SOS1 at serine 1044 in the C-terminal domain to relieve SOS1 autoinhibition. Activated SOS1 transports Na^+^ from the cytoplasm to the apoplast to relieve salt stress (Quintero et al., 2011).

The Na^+^/H^+^ antiporter SOS1 is a key component of Na^+^ efflux from the cytoplasm to the apoplast. The knockout mutant *sos1-1* is highly sensitive to salt stress (Shi et al., 2000; Qiu et al., 2002; Quintero et al., 2002). In Arabidopsis, salt induces phospholipase D activity to hydrolyze phospholipids into PA. PA then binds to MITOGEN-ACTIVATED PROTEIN KINASE KINASE 7 (MKK7) and MKK9, increasing their kinase activity. Downstream MITOGEN-ACTIVATED PROTEIN KINASE 6 (MPK6) is then phosphorylated, and activated MPK6 phosphorylates and activates the SOS1 antiporter to export Na^+^ from cells (Shen et al., 2019). Under normal conditions, SOS1 activity is inhibited by the clade D protein phosphatase 2Cs (PP2C) PP2C.D6 and PP2C.D7. Under salt stress, however, SCaBP8 locates to the plasma membrane and inhibits PP2C.D6 and PP2C.D7 phosphatase activity. PP2C.D6 is released into the cytoplasm, and inhibition of SOS1 by PP2C.D6 and PP2C.D7 is abolished, allowing SOS1 to export Na^+^ from the cytoplasm (Fu et al., 2022). The Ca^2+^ sensor SOS3/CALCINEURIN B-LIKE PROTEIN 4 (CBL4) triggers basal salt tolerance through the SOS3/CBL4–SOS2/CBL-INTERACTING PROTEIN KINASE 24 (CIPK24)–SOS1 pathway, while another Ca^2+^ sensor, CBL8, triggers the CBL8–SOS2/CIPK24–SOS1 module in response to severe salt stress (Steinhorst et al., 2022). The mechanism for sensing mild salt stress may differ from that for sensing severe salt stress in plant cells.

The kinase activity of SOS2 is controlled by several key regulators, such as the PP2C ABA INSENSITIVE 2 (ABI2), GIGANTEA (GI), 14-3-3 proteins, SOS2-LIKE PROTEIN KINASE 5 (PKS5), and BRASSINOSTEROID-INSENSITIVE 2 (BIN2) (Kim et al., 2013; Zhou et al., 2014; Yang Z. et al., 2019; Duscha et al., 2020; Li et al., 2020). ABI2 interacts with SOS2 through the protein phosphatase interaction motif of SOS2. This interaction is disrupted in *abi2-1* mutants, which are more tolerant to salt stress than wild-type plants (Ohta et al., 2003). Interaction of ABI2 with SOS2 therefore negatively regulates salt tolerance. The flowering-time protein GI also negatively regulates salt tolerance. In the absence of salt stress, GI confines SOS2 to the cytoplasm and nucleoplasm, inhibiting its activation of SOS1. Under salt stress, GI is degraded by the 26S proteasome, releasing SOS2 to activate SOS1 to export Na^+^. Meanwhile, flowering is also delayed. GI therefore balances floral transition and salt stress adaption in Arabidopsis (Kim et al., 2013). Similarly, 14-3-3 proteins interact with SOS2, inhibiting SOS2 activity. Salt stress decreases the interaction between 14-3-3 proteins and SOS2, releasing SOS2 and thus activating SOS1 activity (Zhou et al., 2014). Interaction between 14-3-3 proteins and SOS2 is promoted by phosphorylation of SOS2 at serine 294 by PKS5. Salt stress also induces an interaction between 14-3-3 proteins and PKS5, which inhibits the kinase activity of PKS5 and retards the inhibition of SOS2 (Yang Z. et al., 2019). These regulators are involved in the salt stress response phase. Li et al. (2020) identified a molecular switch in the recovery phase following salt stress. After salt stress, SOS3 and SCaBP8 perceive a Ca^2+^ signal and promote BIN2 relocation to the plasma membrane. BIN2 then phosphorylates and inhibits SOS2 to repress the salt stress response and enhances plant growth by releasing the transcriptional activity of BRASSINAZOLE RESISTANT 1 (BZR1) and BRI1-EMS-SUPPRESSOR 1 (BES1). BIN2 therefore functions as a molecular switch between the salt stress response and recovery of growth after salt stress (Li et al., 2020). Recently, evidences showed that light regulates plants’ response to salt stress. Arabidopsis are more tolerant to salt stress under light conditions than under dark conditions. In the light, phytochrome A and phytochrome B (phyA and phyB) translocate into nucleus, interacts with SOS2 and promote SOS2 kinase activity. In nucleus, highly activated SOS2 mediates phosphorylation and degradation of PIF1 and PIF3, leading to alleviate their inhibition of salt tolerance. Besides, light-activated phyA and phyB also enhance SOS2 activity in cytosol, thus enhancing SOS1 activity on the plasma membrane (Ma et al., 2023).

PM H^+^-ATPase is another key regulator of Na^+^ efflux. PM H^+^-ATPase belongs to the P-type subfamily of H^+^-ATPase and export cytoplasmic protons into the apoplast, generating proton gradients and electrical potential differences (Palmgren, 2001; Falhof et al., 2016). SOS1-mediated Na^+^ transport is driven by such proton gradients (Qiu et al., 2002; Yang Y. et al., 2019; Li et al., 2022). Na^+^ exclusion capability of plants generally attenuated under saline-alkali stress due to the influence of high pH on the proton motive force. Thus, H^+^-ATPase activity is a primary determinant of plant saline-alkali tolerance. The Arabidopsis PM H^+^-ATPase PLASMA MEMBRANE PROTON ATPASE2 (AHA2) interacts with the 14-3-3 class of chaperones, which alleviates AHA2 autoinhibition and activates AHA2 (Fuglsang et al., 1999; Fuglsang et al., 2007). Under normal conditions, the SCaBP1-PKS5, SCaBP1-PKS24, and SCaBP3-PKS5 modules phosphorylate Ser-931 of AHA2, which inhibits the interaction between AHA2 and 14-3-3 proteins, thereby repressing its H^+^-ATPase activity, and consequently inhibiting SOS1 activity (Fuglsang et al., 2007; Lin et al., 2014; Zhou et al., 2014; Yang Y. et al., 2019). Under salt stress, ([Ca^2+^]_cyt_) increases. This Ca^2+^ signal is decoded by 14-3-3 proteins, which interact with and repress PKS5 to activate PM H^+^-ATPases, improving SOS1 activity (Yang Z. et al., 2019). DNAJ HOMOLOG 3 (J3) also interacts with PKS5 under salt stress to inhibit its kinase activity and relieve repression of PM H^+^-ATPases. J3 loss-of-function mutants have lower PM H^+^-ATPase activity than wild-type plants and display hypersensitivity to saline and alkaline stresses (Yang et al., 2010). SCaBP3 separates from PM H^+^-ATPases when it senses Ca^2+^ signals triggered by saline-alkali stress. Activity of the SCaBP3-PKS5 complex and phosphorylation at Ser-931 of AHA2 decrease, allowing AHA2 to be activated by 14-3-3 proteins, subsequently enhancing PM H^+^-ATPase activity (Yang Y. et al., 2019). Acetylation of lysine 56 of GENERAL REGULATORY FACTOR 6 (GFR6)/14-3-3λ also inhibits AHA2 activity, making plants hypersensitive to alkali stress (Guo et al., 2022). Moreover, lipids dynamically change under saline-alkali stress (Tasseva et al., 2004). Phosphatidylinositol (PI) inhibits PM H^+^-ATPase activity under normal conditions. Under salt stress, PHOSPHOINOSITIDE 4-KINASE (PI4K) converts PI to phosphatidylinositol 4-phosphate (PI4P), which relieves the inhibition of PM H^+^-ATPase and activates the PM Na^+^/H^+^ antiporter SOS1 to transport Na^+^ out of cells and maintain ion homeostasis (Yang et al., 2021). Recently, a Ca^2+^-dependent signaling pathway to enhance H^+^-ATPase activity and saline-alkali stress tolerance is found in wheat. The researchers found that PM H^+^-ATPase activity is highly related to alkali tolerance in the wheat Shanrong 4 (SR4) variety. Alkali stress induces the expression of Ca^2+^-binding protein C-TERMINAL CENTRIN-LIKE DOMAIN CONTAINING PROTEIN 1 of *Triticum aestivum* (TaCCD1), TaCCD1 interacts with SMALL AUXIN UP RNA215 (TaSAUR215) and enhances the inhibitory effect of TaSAUR215 on two D-CLADE TYPE 2C PROTEIN PHOSPHATASES (TaPP2C.D1 and TaPP2C.D8), which can directly dephosphorylate Thr947 in H^+^-ATPase. Ultimately, the activity of PM H^+^-ATPase TaHA2 is enhanced and alkali stress tolerance is promoted (Cui et al., 2023). Besides, transcriptional regulation also plays important roles in stimulating PM H^+^-ATPase activity under salt stress (Wang et al., 2013; Yao et al., 2019). *Populus euphratica* transcription factor WRKY1 was found to bind the promotor of PM H^+^-ATPase gene *PeHA1* to enhance the *PeHA1* gene expression and salt tolerance (Yao et al., 2019). Phospholipase Dδ in *Populus euphratica* (PePLDδ) is a key enzyme in producing signaling molecules phosphatidic acid (PA). Overexpression of *PePLDδ* in Arabidopsis increases the transcription of *AtSOS1* and PM H^+^-ATPase *AtAHA2*, leading to enhanced Na^+^ exclusion under salt stress (Zhang et al., 2022). Transcriptional regulation of PM H^+^-ATPases provides an important method for saline-alkaline tolerant crop breeding.

## 5 Compartmentation of Na^+^ in vacuoles

Compartmentalizing Na^+^ into vacuoles is an important mechanism for reducing the cytoplasmic Na^+^ concentration under saline-alkali stresses. Vacuolar Na^+^/H^+^ exchangers (NHXs), driven by the proton gradient generated by vacuolar H^+^-ATPase and H^+^-pyrophosphatase, are key components for Na^+^ compartmentation in vacuoles (Drozdowicz and Rea, 2001; Brini and Masmoudi, 2012). The NHX family is divided into two classes. Class I comprises NHX1–NHX4 in Arabidopsis, all localized in vacuoles (Yokoi et al., 2002; Pardo et al., 2006). Overexpressing tonoplast-localized *NHX1* in Arabidopsis increases salt tolerance and Na^+^ accumulation under salt stress (Apse et al., 2003). Similar to the plasma membrane Na^+^/H^+^ exchanger SOS1, tonoplast NHXs in Arabidopsis are activated by SOS2 (Qiu et al., 2004). SCaBP8 interacts with SOS2 to activate Na^+^ sequestration (Kim et al., 2007). However, the Na^+^ content in leaf tissue of *nhx1 nhx2* double mutants under salt stress is higher than that in wild-type plants, while the vacuolar K^+^ content is lower. These results indicate that NHX1 and NHX2 mainly function as K^+^/H^+^ exchangers in the Arabidopsis vacuole (Barragán et al., 2012; Bassil et al., 2012; Bassil and Blumwald, 2014). It seems that other Na^+^ transporters mediate Na^+^ influx into the vacuole from the cytoplasm in Arabidopsis, or that the NHX1 and NHX2 K^+^ transport activity might be mediated by Na^+^ concentration in the cytosol.

Vacuolar H^+^-ATPase is the major H^+^ pump that transports protons, generating a proton gradient and electrical potential to drive secondary active transport of various ions. Under salt stress, SOS2 directly interacts with vacuolar H^+^-ATPases and enhances their H^+^ transport activity to increase the driving force for Na^+^ compartmentation into the vacuole (Batelli et al., 2007).

In addition to Na^+^, Ca^2+^ is also transported into vacuoles under salt stress. CALCIUM EXCHANGER 1 (CAX1) and CAX3 are vacuolar H^+^/Ca^2+^ transporters localized on the tonoplast. *CAX1* is mainly expressed in leaves, while *CAX3* is mainly expressed in roots (Cheng et al., 2005). CAX1-like chimeric transporters are activated by SOS2. Biochemical analysis of Ca^2+^ transport in *cax1* mutants reveals that CAX1 mediates approximately 50% of H^+^/Ca^2+^ transport between the cytoplasm and vacuole; *cax1* and *cax3* mutants display a 38% and 22% reduction in vacuolar H^+^-ATPase activity, respectively. Overexpressing a deregulated version of *CAX1* led to salt sensitivity (Cheng et al., 2005). These results indicate a link between Na^+^ and Ca^2+^ homeostasis in Arabidopsis under salt stress.

## 6 Long-distance ion transportation

At the whole-plant level, inorganic ions, including Na^+^, are absorbed through the root hairs, epidermis, and cortex, then loaded into xylem vessels for transport upward throughout the plant driven by the transpiration pull (Boer and Volkov, 2003; Raddatz et al., 2020). Thereafter, ions are unloaded by shoot or leaf xylem parenchyma cells and transported into phloem sieves (Sunarpi et al., 2005). SOS1 activity during xylem loading and HKT1 activity during xylem unloading might coordinately control the amount of Na^+^ that is eventually exported from roots to shoots.

In addition to Na^+^ exclusion, SOS1 also functions in controlling long-distance Na^+^ transport from roots to shoots to protect roots from salt-induced damage. In Arabidopsis, *SOS1* is preferentially expressed in epidermal cells at the root tip and in parenchyma cells at the xylem/symplast boundary of roots, stems, and leaves (Shi et al., 2002). Na^+^ content analysis of shoots in the *sos1* mutant under mild salt stress (25 mM NaCl) and under severe salt stress (100 mM NaCl) indicates a role for SOS1 in retrieving Na^+^ from xylem sap under severe salt stress, whereas it may function in loading Na^+^ into the xylem under mild salt stress (Shi et al., 2002). The rice *sos1* loss-of-function mutant also shows sensitivity to salt, caused by excessive Na^+^ intake and disturbed Na^+^ loading into the xylem (El Mahi et al., 2019).

Arabidopsis HKT1 in xylem parenchyma cells has a key role in Na^+^ unloading from xylem vessels, thus protecting Arabidopsis leaves from Na^+^ stress. *hkt1* mutants have increased Na^+^ concentration in the xylem sap and reduced Na^+^ concentration in phloem sap. However, the K^+^ content of the xylem is not dramatically influenced (Sunarpi et al., 2005). Under salt stress, *hkt1* mutants show hypersensitive leaves and Na^+^ overaccumulation in xylem sap and shoots, with concomitant Na^+^ reduction in roots. These results indicate that HKT1 selectively transports Na^+^ from xylem vessels to xylem parenchyma cells. Overexpressing *HKT1* in Arabidopsis root stele reduces Na^+^ accumulation in the shoot and enhances salt tolerance (Berthomieu et al., 2003; Møller et al., 2009). In line with this, salt-tolerant rice cultivars accumulate less Na^+^ in leaves and shoots compared with salt-sensitive rice cultivars (Ren et al., 2005), and Na^+^ accumulation in shoots is negatively correlated with salt tolerance in rice (Coskun et al., 2013).

**Figure 1:**
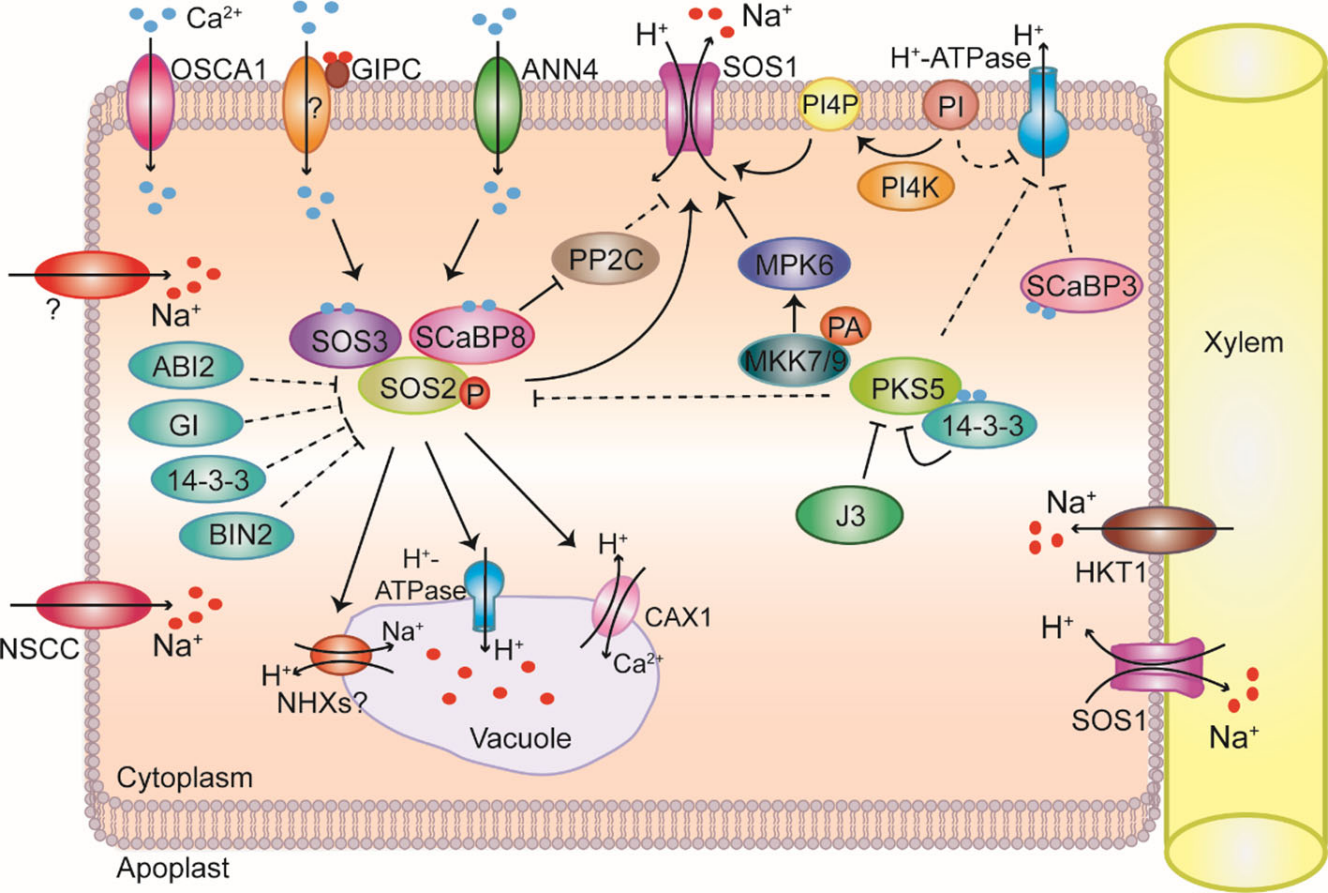
Overview of Na^+^ homeostasis in plant cells under salt stress. Na^+^ (red dots) enters plant cells through nonselective cation channels (NSCC) and unknown transporters. Na^+^ is sensed via an unknown sensing mechanism. The REDUCED HYPEROSMOLALITY-INDUCED [CA^2+^] INCREASE 1 (OSCA1) calcium-permeable channel on the plasma membrane is required for osmotic-stress-induced Ca^2+^ (blue dots) signaling. Na^+^ binds to glycosylinositol phosphorylceramide (GIPC) to gate Ca^2+^ influx channels and induce downstream responses to salt stress. The Ca^2+^-permeable transporter ANNEXIN4 (ANN4) generates Ca^2+^ signals that activate the salt overly sensitive pathway under salt stress. SALT OVERLY SENSITIVE 3 (SOS3) and SOS3-LIKE CALCIUM-BINDING PROTEIN 8 (SCaBP8) perceive salt-induced Ca^2+^ signals to release self-inhibition of SOS2 and recruit SOS2 to the plasma membrane where it becomes active. SOS2 then phosphorylates SOS1, relieving SOS1 autoinhibition. Activated SOS1 transports Na^+^ from the cytoplasm to the apoplast. SCaBP8 locates to the plasma membrane and inhibits the activities of the protein phosphatase 2Cs (PP2C) PP2C.D6 and PP2C.D7 under salt stress, thereby abolishing inhibition of SOS1 by PP2C.D6 and PP2C.D7. Phosphatidic acid (PA) binds to MITOGEN-ACTIVATED PROTEIN KINASE KINASE 7 (MKK7) and MKK9, increasing their kinase activity. MKK7 and MKK9 then activate MITOGEN-ACTIVATED PROTEIN KINASE 6 (MPK6), which phosphorylates and activates the SOS1 antiporter. SOS2 kinase activity is regulated by the PP2C ABA INSENSITIVE 2 (ABI2), GIGANTEA (GI), 14-3-3 proteins, BRASSINOSTEROID-INSENSITIVE 2 (BIN2), and SOS2-LIKE PROTEIN KINASE 5 (PKS5). Increased Ca^2+^ signals are decoded by 14-3-3 proteins, which interact with and repress PKS5 to activate plasma membrane H^+^-ATPases (PM H^+^-ATPases), improving SOS1 activity. DNAJ HOMOLOG 3 (J3) also interacts with PKS5 under salt stress to inhibit its kinase activity and relieve repression of PM H^+^-ATPases. SCaBP3 separates from PM H^+^-ATPases upon sensing Ca^2+^ signals triggered by saline-alkali stress, allowing PM H^+^-ATPases to be activated by 14-3-3 proteins. Na^+^ is compartmentalized in the vacuole through as-yet-unidentified transporters. Na^+^/H^+^ exchanger (NHX) proteins might function as Na^+^/H^+^ exchangers, but this has not been confirmed. In addition to Na^+^, Ca^2+^ ions are also transported into vacuoles through CALCIUM EXCHANGER 1 (CAX1) under salt stress. SOS2 interacts with vacuolar H^+^-ATPases to generate an increased driving force for compartmentalizing Na^+^ into the vacuole under salt stress through putative vacuolar H^+^/Na^+^ and H^+^/Ca^2+^ transporters. SOS1 also functions in controlling long-distance Na^+^ transport from roots to shoots to protect roots from salt-induced damage by loading Na^+^ into the xylem. HIGH-AFFINITY K^+^ TRANSPORTER 1 (HKT1) in xylem parenchyma cells mediates Na^+^ unloading from xylem vessels, thus protecting Arabidopsis leaves from Na^+^ stress.

In summary, to protect roots under salt stress, SOS1 exports Na^+^ out of roots and improves Na^+^ loading into the xylem. Meanwhile, HKT1 transports Na^+^ from xylem vessels into xylem parenchyma cells to protect leaves and shoots (Ali et al., 2021). Therefore, SOS1 and HKT1 antagonistically control the amount of Na^+^ in roots and shoots, and their activities are fine-tuned to avoid futile cycles of Na^+^ loading and unloading (**Figure 1**).

## 7 Saline-alkali-tolerant genes identified by GWAS

In order to improve saline-alkali tolerance in crops, many saline-alkali-tolerant genes or locus were identified through Genome-wide association study (GWAS) analysis. *Na^+^ Content under Saline-Alkali Condition (ZmNSA1)* was identified in by GWAS analysis in natural maize inbred lines with variations of sensitivity to saline-alkali stress (Cao et al., 2020). Under saline-alkaline condition, Ca^2+^ binds to EF-hand domain of ZmNSA1 and triggers its degradation, then increases the transcription level of PM-H^+^-ATPases. Increased abundance of PM-H^+^-ATPases enhance H^+^ efflux and SOS1-mediated Na^+^ efflux from roots, which finally promoting saline-alkali tolerance (Cao et al., 2020). Recently, *Alkaline tolerance 1 (AT1)* in *Sorghum bicolor*, has been identified as a negative regulator of alkali tolerance by GWAS analysis (Zhang et al., 2023). *AT1* encodes an atypical G protein γ subunit, which inhibits the phosphorylation of aquaporins PIP2s (Plasma membrane intrinsic protein 2s) to regulate the export of hydrogen peroxide, and then modulate ROS homeostasis under alkaline stress (Zhang et al., 2023). Knockout of *AT1* increased alkali tolerance and production of sorghum, millet (*Setaria italica*), rice (*Oryza sativa*), and maize (*Zea mays*) (Zhang et al., 2023). These results indicate *AT1* with a great applicational potential in genetic engineering of alkali-tolerant crops.

## 8 Conclusion and perspectives

Saline-alkali stress severely affects plant growth and crop yield, posing a huge threat to agriculture and world food security. Understanding the mechanisms of plant responses to saline-alkali stress is critical for genetically engineering tolerance to saline-alkali stress in crops. Key components that involve in saline-alkali stress responses have been identified over the past few decades, with the mechanisms of ion and pH homeostasis studied in depth. However, some important questions remain. Here we discuss three aspects that need further research.

### 8.1 Mechanism of ion and pH homeostasis under saline-alkaline stress

1) The sodium-sensing mechanism needs to be identified. The cell-surface peptide-receptor complexes RGF1-RGFR and Pep1-PEPR have been identified as pH sensors that function in extracellular pH-mediated growth and immunity in the root apical meristem (Liu et al., 2022). However, it is unknown whether high pH during alkali stress is also sensed by plant membrane RLKs. 2) Studying the specific influence of Na^+^ on plant growth requires disentangling of osmotic and Na^+^ stresses. 3) The mechanisms of plant responses to mild salt stress may be different from those to severe salt stress. 4) Most studies have focused on salt stress or alkali stress alone, with only a few assessing saline-alkali mixed stress. Whether the combination of salt and alkali stresses necessitate distinct response mechanisms in plants is an interesting question.

### 8.2 Crop breeding for saline-alkali tolerance

As research progressed, more key regulators in plant responses to saline-alkaline stress have been identified. Overexpressing positive regulators or knockout negative regulators to saline-alkaline stress has been a common method to genetically improve plant tolerance. However, the balance between plant growth/development and saline-alkaline tolerance should be considered. TOR kinase and ABA receptor balances plant growth and stress response (Wang et al., 2018). Receptor-like kinase FERONIA (FER) coordinates plant growth and salt tolerance via the phosphorylation of phytochrome B (phyB) (Liu et al., 2023). Genes not only enhancing stress tolerance but also improving plant growth, or at least not impacting to plant growth would be an important selection criterion for genetic engineering. The *AT1* nonfunctional mutants, either natural varieties or gene editing mutants in sorghum, millet, rice, and maize, can improve the alkali tolerance and production when cultivated on sodic lands (Zhang et al., 2023). Therefore, genes like *AT1* could be enormous potential for application in crop breeding for salt and alkali tolerance.

### 8.3 Application of Artificial Intelligence in crop breeding

**Figure 2:**
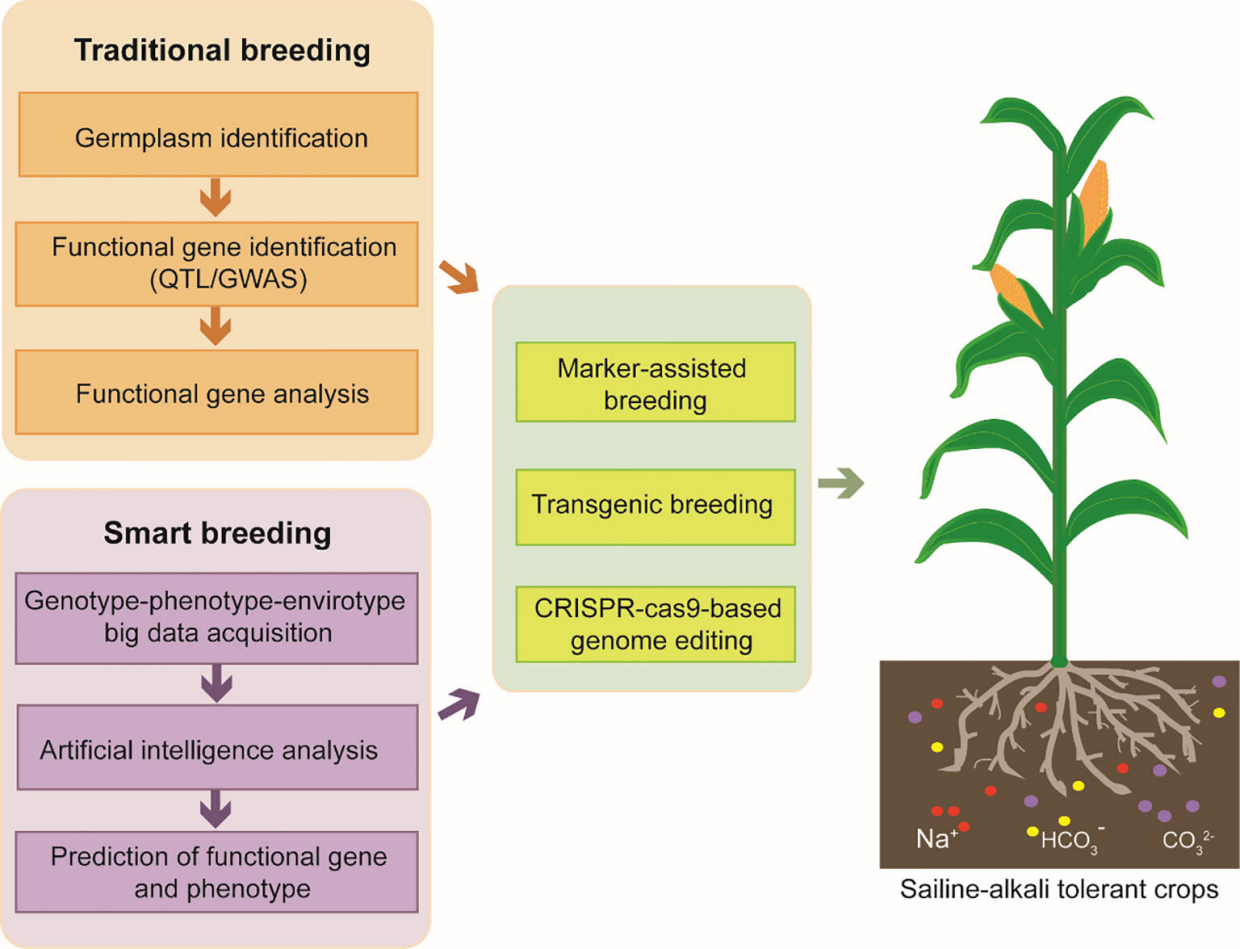
The strategies for improving plant resistance of saline-alkali stress. Traditional breeding requires tolerant/sensitive germplasm identification for saline-alkali stress, key gene identification and gene function analysis. In developing smart breeding, AI could be used in phenotype collecting, phenotype analyzing and phenotype assessment. Genotype-phenotype-envirotype big data could be analyzed by AI algorithm to predict possible gene function and phenotype. Finally, Saline-alkali tolerant crops would be obtained by modern experimental methods, such as marker-assisted breeding, transgenic breeding, CRISPR-Cas9 based genome editing.

Artificial Intelligence (AI) has been a revolutionary technology in many fields and it is also hopeful for promoting crop breeding. The main possible applications of AI in crop breeding contains two major fields, phenomics and genomics. Smart breeding requires gene-phenotype-genomics big data which could be obtained by automatic equipment. Traditional phenotype identification requires a large amount of manpower, funding, time and plentiful breeding experience. AI may contribute in phenotype collecting, phenotype analyzing and phenotype assessment. Although there are many technical difficulties in image acquisition under complex environment and in AI algorithm optimization to analyze massive phenotypic elements, significant achievements have been achieved. Breeding robots and data analysis platforms have been established by several groups or companies, but the scope of application needs to be expanded to more plant species. Genotype-phenotype-envirotype big data is analyzed by AI algorithm to predict possible gene function and phenotype. Saline-alkali tolerant crops would be obtained by experimental methods, such as marker-assisted breeding, transgenic breeding, CRISPR-Cas9 based genome editing (**Figure 2**). As more and more high-throughput data has been obtained through plant multi-omics analysis, AI is more efficient to handle these massive data and screen functional genes to accelerate crop breeding for saline-alkali tolerance.

## Author contributions

YY and JL proposed the concept and content. JL drafted the manuscript. YY revised the manuscript. All authors contributed to the article and approved the submitted version.
